# Microbial-Derived Antioxidants in Intestinal Inflammation: A Systematic Review of Their Therapeutic Potential

**DOI:** 10.3390/antiox14030321

**Published:** 2025-03-06

**Authors:** María José García Mansilla, María Jesús Rodríguez Sojo, Andreea Roxana Lista, Ciskey Vanessa Ayala Mosqueda, Antonio Jesús Ruiz Malagón, Ailec Ho Plagaro, Julio Gálvez, Alba Rodríguez Nogales, María José Rodríguez Sánchez

**Affiliations:** 1 Department of Pharmacology, Centro de Investigación Biomédica (CIBM), University of Granada, 18071 Granada, Spain; garciamansillamariajose@gmail.com (M.J.G.M.); mariajesus.rodriguez.sojo@gmail.com (M.J.R.S.); jgalvez@ugr.es (J.G.); albarn@ugr.es (A.R.N.);; 2 Instituto de Investigación Biosanitaria de Granada (ibs.GRANADA), 18012 Granada, Spain; andreearoxana@correo.ugr.es (A.R.L.); ciskeyay@correo.ugr.es (C.V.A.M.); 3 Instituto de Investigación Biomédica de Málaga y Plataforma en Nanomedicina—IBIMA Plataforma BIONAND, 29590 Málaga, Spain; 4 CIBER de Enfermedades Hepáticas y Digestivas (CIBER-EHD), Instituto de Salud Carlos III, 28029 Madrid, Spain

**Keywords:** dysbiosis, gut, inflammation, microbial-derived antioxidants, microbiome, postbiotics, SCFAs

## Abstract

The potential of microbial-derived antioxidants to modulate intestinal inflammation is increasingly recognized, which is especially important in inflammatory bowel diseases (IBD). Oxidative stress, a major contributor to chronic intestinal inflammation, is the result of an imbalance between the production of reactive oxygen species (ROS) and the body’s antioxidant defenses. This systematic review explores the role of microbial-derived antioxidants in alleviating IBD. Among the main findings are certain compounds, such as exopolysaccharides (EPS) and short-chain fatty acids (SCFAs), which have demonstrated their ability to neutralize ROS and strengthen the integrity of the intestinal barrier, thereby attenuating inflammatory responses. These antioxidants offer the dual benefit of mitigating oxidative stress and rebalancing the gut microbiota, which is often disrupted in IBD. Evidence from preclinical and clinical studies provides a better understanding of the mechanisms involved in the effects of these microbial antioxidants. Conventional treatments for IBD primarily focus on immune modulation. In this context, the integration of microbial-derived antioxidants could offer a complementary approach by addressing both oxidative damage and gut dysbiosis. Further research and clinical trials are essential to establish standardized treatment guidelines and clarify the long-term efficacy of these promising therapeutic agents.

## 1. Introduction

Intestinal inflammation refers to the immune system’s response to harmful stimuli in the gastrointestinal (GI) tract and is central to disorders such as IBD, which includes Crohn’s disease (CD) and ulcerative colitis (UC) [1]. These conditions are characterized by chronic inflammation of the intestine leading to debilitating symptoms such as abdominal pain, diarrhea, fatigue, and malnutrition. Over time, unresolved inflammation can result in serious complications, including intestinal strictures, fistulas, and an increased risk of colorectal cancer [2]. Beyond physical symptoms, the impact on patients’ quality of life is profound, often affecting daily activities and mental health. The underlying mechanisms of intestinal inflammation are complex, involving a combination of immune dysregulation, genetic predisposition, environmental factors, and microbial imbalances. Key to this process is the disruption of the intestinal barrier and the imbalance between pro-inflammatory and anti-inflammatory signals, which perpetuate tissue damage and hinder gut function [3].

A critical factor driving intestinal inflammation is oxidative stress, defined as an imbalance between the production of reactive oxygen species (ROS) and the body’s antioxidant defenses [4]. Excessive ROS generation damages intestinal epithelial cells, disrupts the mucosal barrier, and activates pro-inflammatory signaling pathways, exacerbating the inflammatory response and contributing to chronic disease progression [5]. Oxidative stress also amplifies tissue injury, creating a self-perpetuating loop of inflammation and cellular damage [6,7]. Standard therapies for intestinal inflammation, such as corticosteroids, biologics, and immunomodulators, primarily target immune activation but often fail to address oxidative stress, leaving the epithelial injury unresolved [8,9]. Additionally, these treatments come with drawbacks, such as possible side effects, therapeutic resistance, and incomplete disease remission [8,9,10].

The gut microbiome plays a crucial role in maintaining intestinal homeostasis and immune function. Meanwhile, dysbiosis (an imbalance in the composition of gut microbes) has been closely associated with intestinal inflammation [11]. Pathogenic microbes can exacerbate oxidative stress by producing ROS or interfering with host antioxidant systems, while beneficial microbes contribute to maintaining redox balance, modulating inflammatory responses, and repairing the gut barrier [12,13]. This interplay between the gut microbiome, oxidative stress, and inflammation underscores the potential of microbiome-targeted therapies in managing intestinal inflammatory diseases.

In this context, microbial-derived antioxidants have emerged as a novel therapeutic strategy. These compounds, which are normally produced by gut bacteria or probiotics, offer dual benefits: they neutralize ROS, mitigating oxidative stress, and simultaneously restore microbial balance, enhancing the natural resilience of the gut against inflammation. Examples include exopolysaccharides (EPS), microbial-derived antioxidant enzymes, tryptophan derivatives, and short-chain fatty acids (SCFAs), which are synthesized or secreted by specific microbial strains and exhibit strong antioxidant and anti-inflammatory properties [14,15,16]. In contrast to conventional antioxidants, microbial-derived antioxidants are produced locally, directly addressing the inflamed intestinal environment, ensuring improved bioavailability and targeted effects.

This review aims to systematically assess the role of microbial-derived antioxidants in alleviating intestinal inflammation. For this purpose, we will examine their biochemical properties and mechanisms of action, as well as analyze their efficacy in both experimental and clinical models of inflammatory diseases. Furthermore, throughout the review, the potential of these compounds to counteract and reduce oxidative stress and gut dysbiosis will be analyzed, providing a starting point for the development of innovative therapeutic approaches. By synthesizing available evidence, this review seeks to emphasize the potential of microbial-derived antioxidants as a complementary or alternative strategy to established treatments, targeting the unresolved clinical challenges in the management of intestinal inflammation.

## 2. Materials and Methods

This systematic review was conducted following the Preferred Reporting Items for Systematic Reviews and Meta-Analyses (PRISMA) guidelines to ensure transparency and reproducibility (Supplementary Materials).

### 2.1. Search Strategy

Comprehensive searches were performed across major scientific databases, including PubMed, Scopus, and ScienceDirect, focusing on publications available up to December 2024. The search strategy employed Boolean operators and combinations of key terms such as “microbial-derived antioxidants”, “gut microbiota”, “ROS”, “intestinal inflammation”, “probiotics”, “oxidative stress”, “gut dysbiosis”, “microbial metabolites”, “intestinal barrier”, “IBD”, “SCFA”, and “oxidative defense”. Filters were applied to restrict the results to peer-reviewed articles in English. Initially, the search criteria prioritized studies published within the last five years; however, due to the limited availability of clinical trials on this specific topic, the inclusion window was subsequently broadened.

### 2.2. Study Selection Criteria

The inclusion criteria for this review were designed in order to identify the most relevant and informative studies on the topic. Therefore, only publications written in English and available up to the specified date were considered. Preclinical studies involving animal models of intestinal inflammation were included, particularly those employing chemically induced colitis models as well as genetically modified organisms mimicking IBD. On the other hand, clinical trials and randomized controlled trials examining the efficacy of microbial-derived antioxidants or probiotics capable of producing antioxidants in subjects with IBD were prioritized. Additionally, mechanistic studies based on exploring how gut microbiota or probiotics generate antioxidants and their subsequent effects on oxidative stress, inflammation, and intestinal barrier integrity were included to provide a deeper understanding of the biological processes involved.

With the purpose of refining the scope of the review, exclusion criteria were applied. Studies that focused solely on synthetic, dietary, or plant-derived antioxidants without any connection to microbial activity were excluded, as were articles lacking experimental validation, including opinion pieces, editorials, and theoretical models without supporting data. Research that did not assess key outcomes related to oxidative stress, gut dysbiosis, or inflammation was also excluded, ensuring that only studies with direct relevance to the topic were included in the analysis.

### 2.3. Quality Assessment, Data Extraction and Grouping

The data extraction process was meticulously conducted using a standardized form to maintain consistency and accuracy. Extracted data included the specific types of microbial-derived antioxidants, such as SCFAs, and EPS. Information on the mechanisms of action was also collected, highlighting how these antioxidants neutralize ROS, modulate immune responses, and repair damage to the intestinal barrier. Additionally, key experimental outcomes were recorded, such as changes in ROS levels, concentrations of inflammatory markers like TNF-α and IL-6, shifts in microbiota composition, and improvements in intestinal barrier integrity, including the expression of tight junction proteins. Details of the study designs, including intervention protocols, sample sizes, and treatment time frame, were also documented to enable a comprehensive analysis and comparison across studies.

After applying all the criteria mentioned, duplicates across databases were removed, resulting in the selection of 26 articles for final analysis. The risk of bias was assessed using the Cochrane Risk of Bias tool for randomized controlled trials [17] and SYRCLE’s risk of bias tool for animal studies [18]. Additionally, the quality of evidence was graded using the GRADE framework [18]. This methodical approach enabled the identification of high-quality evidence, providing critical insights into the role of microbial-derived antioxidants in mitigating intestinal inflammation, restoring dysbiosis, and enhancing gut health.

## 3. Results

**Figure 1:**
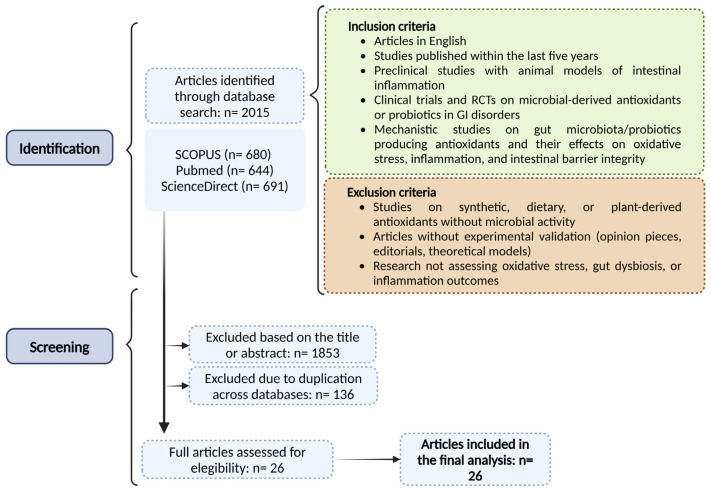
Schematic representation of the systematic review process, detailing the database searches, application of inclusion and exclusion criteria, and final selection of studies. GI, Gastrointestinal; RCT, Randomized Controlled Trial.

A total of 26 studies were included in this systematic review, based on predefined inclusion and exclusion criteria (Figure 1). These studies consisted of preclinical and clinical trials, as well as mechanistic studies, which investigated the effects of microbial-derived antioxidants on oxidative stress, inflammation, and gut dysbiosis. The selected studies employed various experimental designs, including in vitro studies [5,14,16,19,20,21,22,23,24,25] (Table 1), animal models of IBD [15,16,19,22,26,27,28,29,30,31,32,33,34,35,36,37] (Table 2), and human clinical trials [38,39,40] (Table 3), offering a comprehensive evaluation of the impact of microbial-derived antioxidants on gut health and disease.

Among the preclinical studies, a substantial number investigated the effects of microbial-derived antioxidants in animal models of IBD, particularly those induced by chemicals or involving genetically modified organisms that simulate the pathophysiology of CD and UC. A key finding from these studies was the ability of microbial metabolites, such as SCFAs and EPS, to mitigate oxidative stress and reduce inflammation.

## 4. Relevant Sections

### 4.1. Impact of SCFAs on Decreasing Inflammatory Responses and Gut Permeability

SCFAs, and particularly butyrate, were consistently shown to enhance the intestinal barrier function by promoting the expression of tight junction proteins, such as occludin and ZO-1, thus reducing intestinal permeability and preventing further inflammatory responses [19,27,28]. This anti-inflammatory effect is associated with the promotion of regulatory T-cell development and IL-10 production [28], as well as the suppression of neutrophils by inhibiting their migration and reducing cytokine and ROS production [19]. Moreover, according to a study by Xiao et al. (2021), butyrate was shown to alleviate dextran sodium sulfate (DSS)-induced colitis in mice by attenuating the elevated expression levels of TNF-α, IL-6, and IL-12 [29]. This aligns with another study in rats with induced colitis, which further highlights the association between bacterial SCFA production and a reduction in pro-inflammatory cytokines, like TNF-α, IL-6, and IL-1β, and inflammatory mediators, such as iNOS (inducible nitric oxide synthase) and NO (nitric oxide), along with an increase in IL-4 and IL-10 [30].

Clinical data and experimental assays indicate that supplementation with microbial-derived SCFAs led to improved gut microbiota composition, increasing populations of SCFA-producing species, and intestinal barrier function [26,31,32,33,38]. This was correlated with a reduction in gut permeability, which in turn reduced systemic inflammation and improved clinical outcomes in patients with CD [38]. In this sense, it is important to highlight that there is evidence suggesting that an increase in SCFA-producing bacteria (for example, *Roseburia*, *Lachnospiraceae* spp. and *Butyricicoccus*) may enhance the response to other treatments for IBD [27,30,32].

Regarding the effects shown on the imbalance between ROS and antioxidant mechanisms, studies revealed the increase of antioxidant enzymes like glutathione peroxidase (GPx), superoxide dismutase (SOD), and catalase (CAT) associated with SFCA supplementation in murine models of induced colitis [26,27,30]. Consistent with these findings, SCFAs have demonstrated the potential to reverse lipid peroxidation by enhancing the activity of antioxidant enzymes, as reported by Ferrer et al. (2024) [14]. These antioxidant effects, as suggested by recent research, have been associated with the activation of the Nrf2 (nuclear factor erythroid 2-related factor 2) signaling pathway, a key regulator of cellular antioxidant responses [5].

### 4.2. Postbiotics in the Regulation of the Inflammatory Response and Oxidative Stress

A recent study by De Marco et al. (2018) demonstrated that the supernatants of various microbial species, referred to as postbiotics, such as *Lactobacillus casei*, *Lactobacillus acidophilus*, *Lactococcus lactis*, *Saccharomyces boulardii* (SbS), and *Lactobacillus reuteri*, were capable of modulating the inflammatory cytokine profile in human colon-derived cells [23]. The effects of these postbiotics were species-specific, as evidenced by differential cytokine production in macrophages. These findings suggest that the mechanism of action of these metabolites likely involves an initial interaction at the intestinal barrier, followed by subsequent modulation of immune cell activity.

Along the same lines, Qin et al. (2024) reported that postbiotics derived from SbS significantly attenuated intestinal inflammation in DSS-induced colitis models, primarily through the activation of the epidermal growth factor receptor (EGFR) [22]. Notably, these anti-inflammatory effects were further potentiated by thioredoxin, a soluble protein secreted by *Saccharomyces boulardii*. Thioredoxin has been shown to enhance EGFR activation and fortify the intestinal barrier [22].

In the study by De Marco et al. (2019), postbiotics led to a reduction in hydrogen peroxide levels, with the exception of *Saccharomyces boulardii* and *Lactobacillus reuteri*, which did not induce significant changes [23]. Conversely, Qin et al. (2024) demonstrated that postbiotics from SbS exhibited antioxidant properties, a mechanism mediated by thioredoxin [22].

Fermentation by certain probiotic microorganisms releases antioxidant metabolites that can be classified as postbiotics. In this context, organic acids, oxygenated organic compounds, and organoheterocyclics, such as oleanolic acid and kaempferol, as well as fermentation-derived lactic, pentanoic, hexanoic, and isocaproic acids, help mitigate oxidative stress by enhancing the expression of antioxidant enzymes and reducing ROS [5,25].

### 4.3. EPS in Modulating Immunity, Regulating Oxidative Stress, and Supporting Gut Barrier

The use of EPS released from probiotic strains, like *Lactobacillus helveticus*, has shown significant improvements in gut inflammation by enhancing both antioxidant defenses and the intestinal barrier in colitis models in mice [41]. EPS from another probiotic of the same genus, specifically *Lactobacillus rhamnosus*, have also demonstrated potent antioxidant activity in vitro by neutralizing free radicals and protecting intestinal epithelial cells from oxidative stress [21]. Recent studies have reported that EPS directly interact with immune cells, modulating their inflammatory responses and promoting the production of anti-inflammatory cytokines, including IL-10 [36]. The molecular mechanism underlying these results involves the suppression of inflammatory mediators (such as cyclooxygenase-2, IFN-γ, IL-6, IL-1β, IL-12, IL-18, and TNF-α) and the downregulation of nitric oxide synthase expression, thereby mitigating oxidative stress [20,36,41]. It was also reported that EPS inhibited the interaction of lipopolysaccharide (LPS) with toll-like receptor 4 (TLR4), enhancing the anti-inflammatory effect [20]. In addition, these compounds exert a protective effect on the gut epithelium, reinforcing mucosal integrity and preventing further oxidative damage [41]. On the other hand, a study by Ma et al. (2024) indicates a strong synergistic effect between an EPS secreted by *Bifidobacterium longum* subsp. longum XZ01, called BLEPS-1, and *Lactobacillus acidophilus* in a mouse model of DSS-induced UC [35]. The study shows more promising results in relation to inflammation and intestinal barrier regeneration compared to their individual application. These effects include mechanisms such as potentiation of macrophage polarization towards the M2 subtype.

In relation to these findings, a study by Sengül et al. (2011) indicated that the administration of EPS-producing bacteria to rats with experimentally induced colitis using acetic acid was able to reverse oxidative stress [15]. Moreover, similar to the results observed with SCFA application, this microbial metabolite significantly enhanced the activity of several antioxidants enzymes-including SOD, CAT, and both total and reduced glutathione-compared to the control group, while also improving lipid peroxidation. 

### 4.4. Role of Microbial Antioxidants in Modulating Disease Activity of IBD Induced

In recent studies, the supplementation of microbial-derived antioxidants was evaluated due to its potential to alleviate symptoms and improve disease outcomes in patients with IBD [38,39]. Several trials examined the role of probiotics that produce antioxidants, such as *Lactobacillus* and *Bifidobacterium*, in reducing disease activity in cellular and animal models of UC [16,35,36]. In another study, the beneficial effects of a genetically modified *Bifidobacterium bifidum* strain to produce antioxidant enzymes (SOD and CAT) were also observed in cellular colitis models, specifically in the HT-29 cell line, through the regulation of inflammatory cytokines [16]. When the same treatment was applied to a murine model, similar results were obtained, including symptom improvement, antioxidant properties via the upregulation of SOD, GPx, and CAT enzymatic activity, and a protective effect on the epithelial barrier.

Likewise, a randomized controlled trial reported that butyrate supplementation alleviated symptoms in IBD patients (both CD and UC) through regenerative and anti-inflammatory effects [38]. In a similar research, this compound also showed beneficial properties in the treatment of UC patients by alleviating symptoms, maintaining disease remission and reducing levels of calprotectin, an inflammatory biomarker [39]. In addition to the beneficial and antioxidant properties shown by SCFAs, other bacterial metabolites with antioxidant properties have been shown to play an important role in the remission of IBD. Specifically, compounds derived from tryptophan metabolism, such as melatonin, N-acetylserotonin, and 5-OH-tryptophan, have been shown to exert a positive effect on the remission of CD in pediatric patients [40], thus validating and reinforcing the beneficial results obtained by these tryptophan derivatives in preclinical assays [37].

In this context, although most antioxidant compounds are mainly grouped into two large groups, SCFAs and EPS, there are other microbial derivatives that have also been associated with a potent antioxidant activity, such as colipterins derived from *E. coli*, or thioredoxin derived from *Saccharomyces bourlardii*, which have been shown to exert antioxidant and anti-inflammatory effects in in vitro and in vivo assays [22,24].

Analyzed studies provided further insight into the modes of action of microbial-derived antioxidants in intestinal inflammation. These studies highlighted several key mechanisms through which microbial-derived compounds exert their beneficial effects. Notably, antioxidants such as EPS and SCFAs were shown to modulate immune responses by regulating cytokine production and enhancing the activity of antioxidant enzymes like SOD, GPx and CAT [15,26,27,30]. The antioxidant effect of some of these bacterial-derived metabolites are closely associated with the activation of the Nrf2 signaling pathway [5]. Through these mechanisms, microbial antioxidants contributed to a reduction in ROS levels, which in turn mitigated oxidative damage and attenuated the inflammatory cascade [5,16,19,20,22,23,24,32].

Further exploration revealed that microbial antioxidants could restore the balance of the gut microbiome by increasing the abundance of beneficial microbes such as *Roseburia*, *Akkermansia*, and *Lactobacillus* spp. [26,27,34,35,38]. These microorganisms are associated with anti-inflammatory properties and the production of beneficial metabolites, including SCFAs, which contribute to the maintenance of gut health [14,26,27,34]. The restoration of microbiota diversity and the reduction in pathogenic microbial populations were linked to improvements in intestinal inflammation and overall health.

In addition, studies demonstrated that microbial-derived antioxidants influenced mitochondrial function in intestinal cells. This was particularly relevant as mitochondrial dysfunction is commonly associated with oxidative stress in IBD. Microbial antioxidants were shown to improve mitochondrial integrity, reduce mitochondrial ROS production, and enhance cellular energy metabolism, all of which are critical for maintaining intestinal homeostasis and immune function [21,23,27].

## 5. Discussion

**Figure 2:**
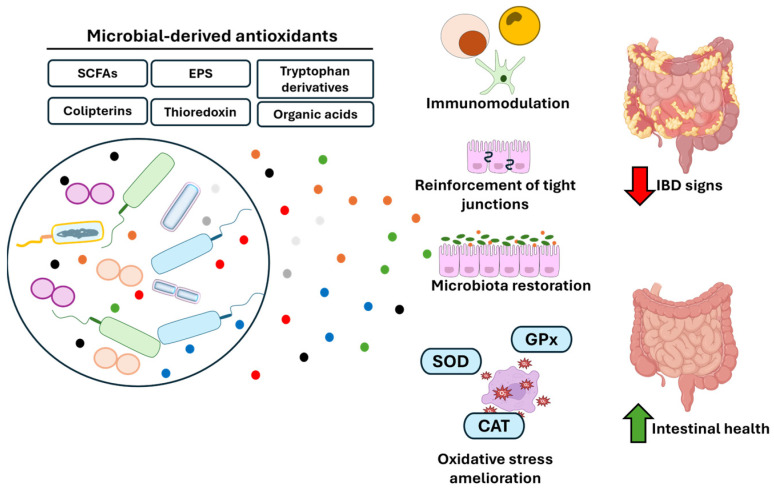
Beneficial effects of microbial-derived antioxidants on gut health. Various metabolites released by bacteria, such as SCFAs, EPS, tryptophan derivatives (e.g., melatonin), organic acids (e.g., lactic acid and isocaproic acid), and other secondary metabolites specific to certain bacteria (e.g., colipterins and thioredoxin), modulate the immune response, strengthen the barrier function, restore the gut microbiome, and reduce oxidative stress. These effects contribute to alleviating IBD symptoms and improving gut health in preclinical and clinical models. CAT: catalase; EPS: exopolysaccharides; GPx: glutathione peroxidase; IBD: inflammatory bowel disease; SCFAs: short-chain fatty acids; SOD: superoxide dismutase.

This systematic review examines the potential of microbial-derived antioxidants in managing oxidative stress and inflammation in IBD. The studies analyzed collectively emphasize the critical role of microbial metabolites in regulating gut homeostasis, reducing oxidative damage, and alleviating intestinal inflammation. Compounds such SCFAs, and EPS have demonstrated robust antioxidant and anti-inflammatory effects, significantly improving disease markers and promoting gut health. Both preclinical and clinical studies support the notion that microbial-derived antioxidants can substantially influence gut health, primarily by reducing ROS levels and enhancing the regeneration of the intestinal barrier. Among these antioxidants, it is necessary to highlight butyrate, which is produced by beneficial bacteria such as *Faecalibacterium prausnitzii* and has been the focus of numerous studies. Butyrate exhibits direct antioxidant properties by targeting important enzymes such as MPO [19]. Additionally, its beneficial effects in IBD are linked to its ability to modulate the immune response, directly interfering with leukocyte migration [19] and with the production of pro-inflammatory cytokines while promoting a regulatory T-cell-mediated response [28]. The positive effects linked to butyrate are also associated with its ability to enhance the epithelial barrier function by upregulating the expression of tight junction proteins [27]. These studies also demonstrate the capacity of butyrate to modulate the gut microbiome by increasing the abundance of beneficial bacterial species, including SCFA-producing bacteria such as *Lachnospiraceae* and *Butyricicoccus* species [33,38]. In this context, growing evidence underscores the crucial role of the microbiome in both the pathophysiology and management of IBD, with microbiome-targeted therapies, such as probiotics, postbiotics, and SCFAs, showing promise in restoring microbial balance and mitigating oxidative stress [14,26]. Across these studies, the restoration of gut microbiota balance, reduction of oxidative stress, and enhancement of intestinal barrier integrity were consistent findings (Figure 2).

Throughout this systematic review, in addition to SCFAs, the other major group of bacterial antioxidant metabolites discussed is EPS. Several studies have evaluated the effects of EPS derived from probiotic bacteria, primarily from the *Lactobacillus* genus, but also from other genera such as *Bifidobacterium*. Various EPS have demonstrated potent antioxidant effects in in vitro and in vivo models of IBD. Although their mechanisms of action depend on the specific type and origin of the EPS, they appear to be associated with the activation of the Nrf2 signaling pathway [21] and the upregulation of host antioxidant enzymes such as CAT, GPx, and SOD [15]. Additionally, this antioxidant effect is linked to an anti-inflammatory response, as EPSs have been shown to block inflammatory signaling pathways activated by pro-inflammatory stimuli like bacterial LPS [20] while increasing the production of anti-inflammatory cytokines such as IL-10 [34,36]. Moreover, certain EPS, such as EPS-1 from *Lactobacillus helveticus* KLDS1.8701, have been shown to strengthen intestinal barrier function and restore gut microbiota balance disrupted in IBD [34,36]. These findings support the beneficial effects of EPS from specific probiotic bacteria and highlight their therapeutic potential in IBD, laying the basis for future clinical trials.

Other postbiotics with antioxidant activity have been highlighted throughout this review. Among them are specific bacterial enzymes [16], tryptophan-derived compounds such as melatonin [37,40], bacterial fermentation products like lactic acid and isocaproic acid [5,25], and secondary metabolites from specific bacterial species, such as colipterins from *Escherichia coli* [24] and thioredoxins from *Saccharomyces boulardii* [22]. All of these compounds have demonstrated antioxidant activity in both in vitro and in vivo, as well as immunomodulatory properties that have resulted in beneficial effects against IBD at both preclinical and clinical levels (Figure 2).

Therefore, the clinical implications of these findings are noteworthy. While conventional IBD treatments, such as corticosteroids, immunomodulators, and biologics, are effective, they often come with significant side effects and limited long-term benefits. In contrast, antioxidant therapies, particularly those targeting the microbiome, present a complementary strategy. Probiotics, postbiotics, and SCFAs, which restore microbial balance and enhance antioxidant defenses in the gut, have been shown to alleviate intestinal inflammation in both animal models and human trials [26,38,40]. Given their ability to directly influence gut microbiota composition and function, these compounds may offer a more targeted and less harmful alternative to traditional therapies.

As discussed, the pathogenesis of IBD is tightly linked to oxidative stress, which amplifies inflammation and damages the intestinal epithelium. ROS are generated in response to inflammatory signals and contribute to a self-perpetuating inflammatory feedback loop, leading to further oxidative damage and immune dysregulation. The reviewed studies provide evidence that antioxidant therapies, particularly those derived from the microbiome, may disrupt this cycle by directly neutralizing ROS, enhancing the activity of antioxidant enzymes like SOD, GPx and CAT, and restoring epithelial barrier function [14,16,26,27,30]. These mechanisms highlight oxidative stress as a promising therapeutic target in IBD, and antioxidant-based therapies could complement traditional immunomodulatory treatments by addressing oxidative damage directly. Although some limitations exist, this review underscores the potential use of these microbe-derived metabolites as a complementary or alternative therapeutic strategy against IBD. Furthermore, unlike other reviews that focus primarily on diet-derived antioxidant compounds, this systematic review highlights the role of metabolites produced by bacteria, particularly butyrate, EPS, and other secondary metabolites derived from tryptophan, as well as specific bacterial compounds such as colipterins and thioredoxin, which have been shown to exert antioxidant effects in various studies discussed throughout this review. The significance of this work lies in synthesizing and compiling these studies, emphasizing the metabolites involved and positioning them as potential targets for future therapies based on these compounds. This review lays the foundation for the development of new preclinical and clinical trials based on exploring the therapeutic potential of these microbial-derived antioxidants.

### Limitations

Despite the insightful findings, this review has certain limitations. First, there is a lack of high-quality, large-scale randomized controlled trials assessing the long-term efficacy of antioxidants in IBD management. Many of the included studies were small-scale or had short durations, limiting the ability to draw definitive conclusions about the lasting impact of antioxidant treatments on gut health and disease outcomes. Furthermore, the heterogeneity of experimental designs, antioxidant measures, and outcome assessments poses challenges in comparing results across studies. Variations in treatment regimens, such as differences in dosage, administration methods, and types of antioxidants used complicate the interpretation of data and make it difficult to establish standardized treatment protocols.

Another significant limitation is the variability in patient populations, particularly in clinical studies. Factors such as individual microbiota composition, genetic predisposition, diet, and lifestyle can all influence how a patient responds to antioxidant therapies, which underscores the need for personalized treatment strategies. The complexity of oxidative stress and its interactions with immune signaling pathways further complicates the interpretation of results, highlighting the need for more targeted research to elucidate the mechanisms by which antioxidants modulate inflammation and oxidative stress in IBD.

## 6. Future Directions

### 6.1. Emerging Therapies Targeting Oxidative Stress and Microbiome Modulation

Given the compelling evidence for the role of antioxidants in managing oxidative stress and gut inflammation, future research should explore novel therapies that activate antioxidant pathways. This includes prebiotics and probiotics that not only restore microbial balance but also enhance the body’s innate antioxidant defenses. The potential for combining these therapies with existing treatments, such as biologics or immunosuppressants, warrants further exploration to assess whether they can enhance therapeutic efficacy and reduce treatment-related side effects.

### 6.2. Need for Long-Term Clinical Studies on Antioxidants

One critical gap in the current literature is the lack of long-term studies on the use of antioxidants in IBD. Most clinical trials have been limited to short-term evaluations, which may not fully capture the long-term effects and safety profiles of antioxidant interventions. Future studies should aim to assess the durability of antioxidant therapy effects, as well as their potential to prevent disease relapse or modulate chronic inflammation over time.

### 6.3. Potential for Personalized Antioxidant Therapy Based on Microbiome Profiles

A promising future direction is the development of personalized antioxidant therapies tailored to individual microbiome profiles. Research into how specific microbiota compositions influence the efficacy of antioxidants could lead to more targeted interventions, optimizing both bioavailability and therapeutic outcomes. Personalized treatment plans could not only improve clinical outcomes but also minimize treatment variability and side effects, offering a more precise and individualized approach to managing IBD.

## 7. Conclusions

This review underscores the significant therapeutic potential of microbiome-derived antioxidants in modulating oxidative stress and inflammation, both key factors in the pathogenesis of IBD. Microbial metabolites such as SCFAs and EPS offer promising alternatives or adjuncts to traditional therapies, providing a dual mechanism of action that targets both inflammation and oxidative damage. However, given the variability in study designs and patient responses, further high-quality, long-term clinical trials are necessary to establish standardized treatment protocols and optimize the efficacy of antioxidant therapies. Ultimately, personalized treatment approaches that leverage microbiome profiling and antioxidant modulation could revolutionize the management of IBD, improving patient outcomes and reducing dependence on conventional immunosuppressive treatments.

## Figures and Tables

**Table 1:** Research findings from recent in vitro studies on the role of postbiotics as novel therapeutic approaches for IBD. BMDM: bone marrow-derived macrophages; CAT: catalase; CD: Crohn’s Disease; DPPH: 1,1-dipheny-l,2-picrylhydrazyl; EPS: exopolysaccharides; GPx: glutathione peroxidase; LPS: lipopolysaccharide; MPO: myeloperoxidase; ROS: reactive oxygen species; SbS: *Saccharomyces boulardii* supernatant; SCFAs: short-chain fatty acids; SOD: superoxide dismutase; UC: ulcerative colitis.

Compound	Findings	Preclinical Study In Vitro		
		Sample	Methodology	Reference
SCFAs	Reduction of oxidative stress - Enhancement of antioxidant enzyme activity - To neutralise lipid oxidation levels	Caco-2/TC7 cell line as an in vitro model of oxidative stress in the intestinal epithelium	Oxidative stress was induced by TNF-α. Secondly, cells were incubated with SCFAs (acetate, propionate, or butyrate), followed by an evaluation of antioxidant enzyme activity and protein and lipid oxidation levels.	[14]
	Butyrate exerts an anti-inflammatory and antioxidant effect by - Inhibiting neutrophil production of cytokines - Suppressing MPO activity and reducing ROS production - Suppressing neutrophil migration.	Neutrophils isolated from peripheral blood of healthy controls (*n* = 8), active CD (*n* = 10) and active UC (*n* = 13)	Neutrophils were isolated from healthy individuals and patients with CD or UC, stimulated with LPS and treated with butyrate. Cytokine and ROS levels, as well as the capacity of migration of neutrophils, was evaluated after butyrate treatment.	[19]
EPS	EPS from *Lactobacillus plantarum* were shown to - reduce the expression levels of pro-inflammatory cytokines and mediators (COX-2 and iNOS) and to regulate inflammatory pathways - inhibit the LPS-TLR4 interaction	RAW 264.7 mouse macrophage cells	Induction of inflammatory response in vitro by LPS and assessment of anti-inflammatory effects on RAW 264.7 mouse macrophage cells pre-incubated with EPS from *Lactobacillus plantarum*	[20]
	- reduce ROS and ferrous ion chelating activity - Improve oxidative stress and cell viability.	IPEC-J2 cell line from porcine intestinal epithelial cells	Evaluation of the anti-apoptotic and antioxidant potential of EPS produced by *Lactobacillus rhamnosus* GG in vitro, including their amelioration of oxidative stress induced in a cell model	[21]
Postbiotics from *Saccharomyces boulardii* supernatant (SbS)	- To mitigate intestinal inflammation through activation of EGFR - Improvement of oxidative stress through thioredoxin secretion	Caco-2 and HT-29 cell lines	Analysis of the role of SbS and thioredoxin to improve colitis in cellular models and assessment thioredoxin modulation of the EGFR pathway in vitro	[22]
Postbiotics from *Lactobacillus casei*, *Lactobacillus acidophilus*, *Saccharomyces boulardii*, *Lactobacillus reuteri*, and *Lactococcus lactis*	- To regulate the inflammatory cytokine profile - Reduction of ROS levels in a dose-dependent manner - To reduce the concentration of hydrogen peroxide	HT-29 cell line (from human adenocarcinoma) Macrophages derived from monocytes stimulated with LPS	Evaluation of the effect of probiotic-derived metabolites in in vitro models of gut inflammation to assess their immunomodulatory role in the HT-29 cell line and macrophages. This included calculating the disease activity index and conducting metagenomic and proteomic studies.	[23]
Microbial-derived antioxidants enzymes: *streptococcal* SOD and *lactobacillus* CAT	- To regulate the inflammatory cytokine profile	HT-29 cell model incubated in LPS	Study of the antioxidant and anti-inflammatory function derived from genetically modified *Bifidobacterium bifidum* strains in a cell model of colitis	[16]
Colipterins	Colipterins exhibited both in vitro radical scavenging activities and upregulation of anti-inflammatory cytokines.	Free radical DPPH, Hydrogen peroxide Mouse BMDM cells and human primary cells	Study of the radical scavenging activity of different colipterins from *Escherichia coli* in different in vitro models that use oxidant species and cells	[24]
Microbial-derived antioxidants produced by probiotic fermentation	Microbial-derived antioxidants mitigated the ROS accumulation and ameliorated the oxidative stress state by enhancing the activity of antioxidant enzymes like SOD, GPx, and CAT.	IPEC-1 intestinal epithelial cells or transfected with NLRP3 siRNA or Nrf2 siRNA	Study of the antioxidant and anti-inflammatory capacity of a mixture of microbial derivatives (including organic acids, organic oxygen compounds organoheterocyclics) benzenoids phenylpropanoids and polyketides) in IPEC-1 cells	[5]
Microbial-derived antioxidant metabolites obtained from fermentation	The fermentation of ginseng extract with probiotic bacteria in the presence of a reconstructed minimal human gut microbiota mediates an antioxidant effect on the HT-29 human intestinal cell line.	HT-29 epithelial cell line	The metabolites derived from the fermentation of the ginseng extract by the probiotics, the minimal core, and the whole gut bacterial community were isolated: lactic, pentanoic, hexanoic and isocaproic acids were identified and cultured. with HT-29 cells to study their impact on ROS production	[25]

**Table 2:** Evidence from preclinical in vivo studies on the effect of gut microbiota-derived antioxidant metabolites as promising treatment for IBD. CAT: catalase; EGFR: epidermal growth factor receptor; DSS: dextran sodium sulphate; GPx: glutathione peroxidase; MDA: malondialdehyde; NO: nitric oxide; SbS: *Saccharomyces boulardii* supernatant; SCFA: short-chain fatty acids; SOD: sodium dismutase; TNBS: Trinitrobenzenesulfonic acid; UC: ulcerative colitis.

Compound	Findings	Preclinical Study In Vivo		
		Sample	Methodology	Reference
SCFAs	Butyrate alleviates colitis by preventing neutrophil recruitment and suppressing their inflammatory and pro-oxidant signaling.	Mouse model of colitis induced by DSS (*n* = 12 per group)	Colitis was induced in C57BL/6J mice by the administration of DSS and the impact of Butyrate on inflammation and oxidative stress was evaluated.	[19]
	- Antimicrobial activity against pathogens - To increase antioxidant enzymes (SOD, GPx and catalase CAT) - To attenuate expression levels of pro-inflammatory cytokines - Mitigate intestinal damage - To alleviate symptoms	Porcine fecal samples (*n* = 10) Mice (*n* = 10 per group)	*Lactiplantibacillus argentoratensis* AGMB00912, isolated from porcine fecal samples, was administered to mice to assess its effects on intestinal health, immune response, and gut microbiota composition by increasing SCFA production.	[26]
	- To enhance SCFA production, with the following effects: - Promotion of the expression of tight junction proteins (occludin and ZO-1) - ROS reduction and increased activity of antioxidant enzymes (including SOD and CAT) - Anti-inflammatory (via cytokine regulation), antioxidant and dysbiosis-enhancing effects - Reduction of colitis symptoms - Preserving the gut barrier integrity.	Mouse model of colitis induced by DSS (*n* = 16 per group)	Administration of *L. hypoglauca* and *S. baicalensis* extracts and evaluation of inflammatory, antioxidant, and colonic microbiota parameters, as well as the disease activity index	[27]
	- To mitigate colitis onset - To promote T-cell regulatory responses - To induce Th1 cell IL-10 production through GPR43	Mouse model of colitis induced by T cell transfer in immunodeficient mice (*n* = 4)	Oral supplementation of butyrate and evaluation of its protective effects against disease progression	[28]
	- Alleviation of colitis symptoms - Reduction of histological colon damage and expression of key pro-inflammatory mediators	DSS-induced murine colitis model (*n* = 6 per group)	Butyrate supplementation and subsequent analysis of disease development	[29]
	Beneficial effects associated with increased SCFA levels are as follows: - Enhancement of antioxidant enzyme activity (SOD, GPx, and CAT) - Reduction in the expression levels of pro-inflammatory cytokines.	Rats with TNBS-induced colitis (*n* = 7 per group)	Fermented okara (a soybean byproduct) treatment and its effects on gut microbiota composition, SCFA levels, and inflammatory and oxidative parameters	[30]
	Butyrate showed improvements in the inhibition of colitis and dysbiosis-induced colitis.	Mice with colitis-associated colorectal cancer (*n* = 9–12 per group)	To evaluate the antitumor potential, as well as its effects on intestinal inflammation and dysbiosis, of sodium butyrate in a colitis-associated colorectal cancer model	[31]
	The administration of nanocapsules with a mix of probiotics induce the production of acetic acid, propionic acid, butyric, isobutyric acid, valeric, isovaleric acid, and total SCFAs. These changes are associated with the following: - A reduction in oxidative stress by reducing MDA and NO levels. - An anti-inflammatory effect by downregulating pro-inflammatory cytokines - Gut dysbiosis restoration - Reinforcement of tight junctions.	Sprague–Dawley rats with colitis induced by DSS administration (*n* = 10 per group)	To study the effect of a nano-encapsulated multi-strain probiotics formulation (*Bifidobacterium breve* DSM24732, *B. coagulans* SANK 70258 and *L. plantarum* DSM24730) on intestinal inflammation and oxidative stress induced by DSS administration in rats	[32]
	Butyrate resulted from the administration of four SCFA-producing bacteria (*Butyricimonas paravirosa*, *Coprococcus comes*, *Megasphaera indica*, *Agathobaculum butyriciproducens*) which protect germ-free mice from DSS-induced colitis by - Increasing mucin thickness - Restoring gut microbiome - Reducing the inflammatory response - Exerting an antioxidant effect by inhibiting MPO and NO prodiction.	C57BL/6 Germ-free mice with DSS-induced colitis (*n* = 4/6 per group) and treated with SCFA-producing bacteria	Administration to 4 different SCFA-producing bacteria to germ-free mice with colitis and evaluation of the immune response, histopathology, SCFA levels and disease indices	[33]
EPS	- To modulate inflammatory response by interacting with immune cells and modulating cytokine production - To maintain the epithelial integrity and enhance the intestinal barrier - To increase SCFAs production - To improve gut microbiota composition	DSS-induced UC mice supplemented with an EPS produced by *Lactobacillus helveticus* KLDS1.8701 (*n* = 12 per group)	Assessment of anti-inflammatory properties of an EPS produced by *Lactobacillus helveticus* KLDS1.8701 in a UC model	[34]
	- BLEPS-1 enhances SCFA production - Synergistic effect of BLEPS-1 and *Lactobacillus acidophilus*: - In regulating inflammation and modulating microbiota composition - On macrophage M2 polarization	DSS-induced colitis mouse model (*n* = 7–8 per group)	Assessment of the potential of BLEPS-1, an EPS produced by *Bifidobacterium longum* subsp. longum XZ01, to promote the growth of *Lactobacillus acidophilus* and its immunological, microbial, and anti-inflammatory effects in colitis	[35]
	- To increase antioxidant enzyme activity (including SOD, GPx, and CAT) - To improve oxidative stress To improve lipid peroxidation	Rat model of induced colitis through acetic acid administration (*n* = 10 per group)	Treatment analysis of EPS-producing bacteria supplementation on intestinal oxidative stress in an animal model of induced colitis, considering lipid peroxidation levels and antioxidant enzyme activities	[15]
	EPS considerably ameliorated colitis by - Restoring the intestinal microbiota composition - Increasing the content of butyric acid - Reducing the production of inflammatory cytokines and enhancing the anti-inflammatories.	Mouse model of induced colitis through the administration of DSS to ICR mice (*n* = 6–8 per group)	EPS produced by *Lactobacillus plantarum* YW11 was administered at different dosages to DSS-induced colitic mice. Inflammatory and oxidative stress states were assessed.	[36]
Postbiotics from *Saccharomyces boulardii* supernatant (SbS)	- To mitigate intestinal inflammation through activation of EGFR and cytokine modulation - Promotion of the expression of tight junction proteins (occludin and ZO-1) - To improve activity disease - Thioredoxin secretion: - enhances cell viability and anti-inflammatory effects - strengthens the intestinal barrier	DSS-induced colitis in mice supplemented with SbS and thioredoxin (*n* = 6–8 per group)	Analysis of the potential role of SbS and thioredoxin in a colitis model and assessment thioredoxin modulation of the EGFR pathway	[22]
Microbial-derived antioxidants enzymes: streptococcal SOD and lactobacillus CAT	- To regulate the inflammatory cytokine profile - To alleviate symptoms - To enhance antioxidant enzymes activity (SOD, GPx and CAT) - To preserve the epithelial barrier by enhancing the expression of tight junction proteins (occludin and ZO-1)	Murine model of DSS-induced colitis (*n* = 8 per group)	Study of the antioxidant and anti-inflammatory function derived from a genetically modified *Bifidobacterium bifidum* strain in murine models of colitis	[16]
Tryptophan metabolites	*Lactobacillus fermentum* 016 supplementation to colitic mice increase the production of tryptophan-derived metabolites (melatonin, kynurenic acid, 3-indoleacetic acid, 5-methoxytryptophan, and 5-hydroxyindoleacetic acid) with antioxidant properties that: - Reduce colonic damage - Improve the mucosal barrier function - Reduce systemic inflammation - Increase the antioxidant effect by decreasing MDA levels and promoting the activity of SOD, CAT and GPx - Modulate gut dysbiosis.	Murine model of DSS-induced colitis (*n* = 12 per group)	Study the effect of administering *Lactobacillus fermentum* 016 in a murine colitis model, focusing on its anti-inflammatory and antioxidant properties, and compare its effects with those of sulfasalazine.	[37]

**Table 3:** Findings from clinical studies on the potential of gut microbiota-derived antioxidant metabolites as promising therapies for IBD. CD: Crohn’s disease; CDED + PEN: partial enteral nutrition; EEN: exclusive enteral nutrition; SFCA: short-chain fatty acids; UC: ulcerative colitis.

Compound	Findings	Clinical Study		
		Sample	Methodology	Reference
Butyrate (SCFA)	- To improve gut microbiota composition in colitis - To increase the abundance of SCFA-producing species	CD patients (*n* = 19) UC patients (*n* = 30) Healthy controls (*n* = 18)	Study of the impact of colonic butyrate administration on the fecal microbiota in IBD patients by a randomized controlled trial	[38]
	- Improvement of remission and symptoms - To decrease fecal calprotectin levels	UC patients (*n* = 39), of whom 18 received butyrate treatment and 21 comprised the control group	Study on the effect of oral butyrate administration in UC patients on symptoms, remission, and inflammatory parameters of the disease (assessed by fecal calprotectin levels, among others)	[39]
Tryptophan-derived metabolites	Specific microbial-derived metabolites with antioxidant capacity, such as melatonin, N-acetylserotonin, and 5-OH-tryptophan, are associated with diet-induced and sustained remission in Pediatric CD.	Fecal samples from pediatric CD patients fed with 2 different nutritional therapies (CDED + PEN: *n* = 22; or EEN: *n* = 21)	A total of 21 tryptophan metabolites were quantified in stool samples from patients enrolled in a prospective randomized trial comparing CDED + PEN and EEN for remission in mild to moderate pediatric CD. Association analyses were conducted to assess the impact of these metabolites on disease remission.	[40]

## Supplementary Materials

The following supporting information can be downloaded at: https://www.mdpi.com/article/10.3390/antiox14030321/s1, PRISMA 2020 Checklist [42].

## Abbreviations

The following abbreviations are used in this manuscript:

CAT	catalase
CD	Crohn’s disease
DSS	dextran sodium sulfate
EGFR	epidermal growth factor receptor
GPx	glutathione peroxidase
IBD	inflammatory bowel disease
Nrf2	nuclear factor erythroid 2-related factor 2
PRISMA	Preferred Reporting Items for Systematic Reviews and Meta-Analyses
ROS	reactive oxygen species
SbS	supernatant from *Saccharomyces boulardii*
SCFA	short-chain fatty acids
SOD	superoxide dismutase
TLR4	toll-like receptor 4
UC	ulcerative colitis
